# Stimulation of Cell Elongation by Tetraploidy in Hypocotyls of Dark-Grown Arabidopsis Seedlings

**DOI:** 10.1371/journal.pone.0134547

**Published:** 2015-08-05

**Authors:** Hideki Narukawa, Ryusuke Yokoyama, Shinichiro Komaki, Keiko Sugimoto, Kazuhiko Nishitani

**Affiliations:** 1 Laboratory of Plant Cell Wall Biology, Graduate School of Life Sciences, Tohoku University, Sendai, Japan; 2 RIKEN Center for Sustainable Resource Science, Yokohama, Kanagawa, Japan; The University of Tokyo, JAPAN

## Abstract

Plant size is largely determined by the size of individual cells. A number of studies showed a link between ploidy and cell size in land plants, but this link remains controversial. In this study, post-germination growth, which occurs entirely by cell elongation, was examined in diploid and autotetraploid hypocotyls of *Arabidopsis thaliana* (L.) Heynh. Final hypocotyl length was longer in tetraploid plants than in diploid plants, particularly when seedlings were grown in the dark. The longer hypocotyl in the tetraploid seedlings developed as a result of enhanced cell elongation rather than by an increase in cell number. DNA microarray analysis showed that genes involved in the transport of cuticle precursors were downregulated in a defined region of the tetraploid hypocotyl when compared to the diploid hypocotyl. Cuticle permeability, as assessed by toluidine-blue staining, and cuticular structure, as visualized by electron microscopy, were altered in tetraploid plants. Taken together, these data indicate that promotion of cell elongation is responsible for ploidy-dependent size determination in the Arabidopsis hypocotyl, and that this process is directly or indirectly related to cuticular function.

## Introduction

Cell proliferation and differentiation, including cell expansion, are crucial processes needed for organogenesis in multicellular organisms. However, compared with animal tissues, organ growth in plants is more heavily dependent on cellular expansion. Plant cells are able to expand dramatically, and can sometimes increase their volumes greater than 1000-fold [1]. The size of the mature plant organ is largely determined by individual cell size. Cell size is regulated by a number of factors, including the timing of cell expansion onset, the cellular expansion rate, and the timing of expansion cessation. The regulatory mechanisms responsible for these processes have been investigated from two main perspectives. One perspective focuses on changes to the mechanical properties of the cell wall. These changes, which affect cellular water uptake, are regulated by environmental cues and plant hormones [2]. The other perspective focuses on autopolyploidy and endopolyploidy, both of which have been correlated with cytoplasmic volume and, hence, cell growth [1].

Endopolyploidy, which is essential for angiosperm development, is controlled by an endoreduplication cycle whereby the genome is duplicated without cellular division. Conversely, autopolyploidy arises spontaneously or experimentally through the production of a 2n gamete during reproduction [3]. The growth phenotype of dwarf mutants defective in endoreduplication can be overcome by colchicine-induced polyploidization [4], and endopolyploidy and autopolyploidy can therefore be considered as comparable to one another with respect to polyploidy-dependent plant size.

Two main hypotheses have been proposed to explain the mechanism by which polyploidy affects cell size [1, 5]. The first of these proposes that gene expression is proportional to ploidy level. In this case, total metabolic activity would be enhanced in polyploid tissues, and this would lead to an increase in growth rate [1]. Circumstantial support for this hypothesis is provided by the observation that endoreduplication commonly occurs in rapidly developing tissues that have high metabolic activity [6]. Further supportive evidence is obtained using microarray analysis that shows only a few differentially expressed genes among different ploidy levels, indicating that the abundance of most of the mRNA may increase in polyploid cells [1, 7]. Recently, Bourdon et al. provided the direct evidence that endopolyploidy increased transcription of rRNA and mRNA on a per-nucleus basis [8]. The second hypothesis envisages the involvement of certain specific genes in ploidy-dependent cell-size determination [5]. This hypothesis is based on the assumption that a cell is a composite of linear, planar, and spherical modules (e.g., microtubule, cell membrane, and cytoplasm, respectively), and that the abundance of individual gene products required for their activities is proportional to their dimension(s). In this model, for the cell volume to double, the transcripts required for spherical modules would need to increase twofold, whereas the transcripts needed for linear and planar modules would need to increase 2^2/3^-fold and 2^1/3^-fold, respectively [5, 9]. This hypothesis is partially supported by transcriptomic data showing that a number of cell-surface localized proteins are significantly downregulated in tetraploid yeast [10]. In land plants, however, no genes with specific functions shared between different species have yet been identified as being altered according to ploidy level. The mechanisms by which ploidy levels affect cell size therefore remain elusive.

Cells elongate more than 100-fold during etiolated development in *Arabidopsis thaliana* hypocotyls. As no cell division occurs, post-germination hypocotyl growth is governed exclusively by cell expansion, and *A*. *thaliana* hypocotyls therefore serve as useful model systems for the study of cell size. Despite the lack of apparent mitotic activity during post-germination growth, a large portion of cells in *A*. *thaliana* hypocotyls undergo endoreduplication. In some experiments, the level of endoreduplication was higher in dark-grown hypocotyls than in light-grown ones, which may be indicative of a positive correlation between ploidy level and plant size [11]; however, other similar research found no such correlation [1, 12]. This suggests that factors other than ploidy are important in determining cell size. It remains possible that the correlation between ploidy and cell size occurs due to mutual feedback between the two processes, and that ploidy is not the main driving force behind cell growth [6, 13]. The role of ploidy in determining cell size remains controversial [12].

In this study, we wished to examine more closely the relationship between ploidy and cell growth. Cell size is determined by a range of factors that vary temporally and spatially according to developmental stage and tissue type. It seems unlikely, therefore, that a common signaling pathway is shared by all plant species and tissue types. We therefore focused on cell elongation occurring in a specific region of the hypocotyl of dark-grown *A*. *thaliana*, and compared growth parameters and gene expression profiles between diploid and tetraploid plants.

## Materials and Methods

### Plant materials and growth conditions

*Arabidopsis thaliana* (L.) Heynh. Col-0 plants were used as a diploid control. Tetraploid plants were generated in the Col-0 background, and were assessed for the ploidy using both flow cytometry and fluorescence in situ hybridization for centromeres as described by Breuer et al. [4]. Arabidopsis seeds were surface-sterilized in 70% ethanol and were sown on Murashige and Skoog (MS) medium, adjusted to pH 5.8, containing 2% (w/v) sucrose and 0.4% gellan gum. Following storage at 4°C for 3 days, seeds on MS medium were germinated in continuous white light (45 μmol m^-2^s^-1^) at 22°C in a growth chamber (Nippon Medical and Chemical Instruments Co., Ltd., Osaka, Japan). For light incubation, geminated seeds were allowed to grow under the same light conditions with the MS medium plate placed horizontally. For dark incubation, seeds were germinated under light conditions for 1 day, but MS plates were subsequently double-wrapped in aluminum foil and placed vertically to allow seedlings to grow along the surface of the medium.

For incubation in light with a low red/far-red ratio, seedlings were irradiated with 0.4 μmol m^-2^s^-1^ white light supplemented with 5.5 μmol m^-2^s^-1^ far-red light (730 nm light-emitting diode).

### Hypocotyl and cell-length measurements

Light-grown seedlings were imaged using a microscope (Leica M205 FA, Leica Microsystems, Heerbrugg, Switzerland). Whole dark-grown seedlings were imaged on the MS plates at 1-day intervals using a scanner (GT-9800F, Epson Japan) at a resolution of 400 dpi. Hypocotyl cortical cells were imaged using a microscope (DMRXP, Leica Microsystems). ImageJ 1.44 software (www.rsb.info.nih.gov/ij; Wayne Rasband, National Institutes of Health) was used to determine hypocotyl and cell lengths from digital microscopy and scan images.

To measure growth rates in the elongation zone of dark-grown hypocotyls, the upper part of the hypocotyl was divided into several ~1 mm subsegments by application of lanolin marks on the surface. The growth rate for each of the marked segments was measured by recording the distance between lanolin marks on images scanned at 720 dpi (GT-9800F).

### RNA extraction and microarray analysis

Total RNA samples were separately extracted from three batches of tissue segments derived from the apical 4 mm region of 7-day-old dark-grown hypocotyls of diploid (wild-type) and tetraploid Arabidopsis using a Qiagen RNeasy plant mini kit (Qiagen Japan, Tokyo, Japan). RNA samples were treated with TURBO DNase (Ambion Japan, Tokyo, Japan), and labeled with a Low Input Quick Amp labeling kit two-color (5190–2306; Agilent Technologies) and Agilent RNA Spike-In kit two-color (5188–5279; Agilent Technologies). The cRNAs of diploid and tetraploid samples were labeled with Cy3 and Cy5 fluorescent dyes, respectively. Microarray analysis was performed by hybridizing three arrays in a single Arabidopsis (V4) Gene Expression Microarray slide (4×44 K; Agilent Technologies) with mixture of the Cy3 and Cy5 labeled cRNAs. After hybridization by Gene Expression Hybridization kit (5188–5242; Agilent Technologies), the microarray slide was scanned using a scanner model G2539A with scan control A.8.5.1(Agilent Technologies).

Data analysis was performed using Feature Extraction 10.10.1.1 (Agilent Technologies) and GeneSpring GX 12.6.1 (Agilent Technologies). The *p* value of each gene was calculated by *t*-test against zero, and corrected by the Benjamini and Hochberg false discovery rate (FDR) method [14]. Differential expression between diploid and tetraploid seedlings was considered to be statistically significant at *p* < 0.05 and FDR < 0.1. All the microarray data were deposited at NCBI Gene Expression Omnibus (GOE) database (http://www.ncbi.nlm.nih.gov/geo/) under the GEO accession number GSE69349.

DAVID bioinformatics resources 6.7 were used for Gene Ontology analysis [15] with a cutoff value < 0.05 for the Benjamini-corrected *p*-value of Fisher’s exact test.

### Quantitative Real time RT-PCR analysis

Total RNA samples from the apical 4 mm region of 7-day-old dark-grown hypocotyls of diploid and tetraploid Arabidopsis were treated with TURBO DNase (Ambion Japan, Tokyo, Japan). One-step real time RT-PCR was performed using MultiScribe reverse transcriptase (Applied Biosystems), SYBR Green PCR Master mix (Applied Biosystems) and 7300 Real Time PCR System (Applied Biosystems). Primer sets used for PCR reaction are listed in S1 Table.

### Cuticle analysis

Hypocotyls were stained using toluidine-blue as described by Tanaka et al. [16], and images were recorded using a digital camera (Canon Powershot G12, Canon Inc.,Japan)

### Transmission electron microscopy (TEM) analysis

For TEM analysis of cuticles, the apical 3 mm regions of 7-day-old dark-grown hypocotyls from diploid and tetraploid plants were excised and fixed at 4°C overnight in 0.05 M cacodylate buffer (pH 7.4) containing 2% glutaraldehyde and 2% paraformaldehyde. Samples were next fixed with 1% tannic acid in cacodylate buffer for 1 h at 4°C and were then rinsed four times in 0.05 M cacodylate buffer (30 min per wash). Finally, samples were fixed in 1% osmium tetroxide for 3 h at 4°C. After dehydration in graded ethanol solutions (50%, 70%, 90%, and 100%), samples were embedded in epoxy resin Quetol-651 (Nisshin EM Co. Ltd., Japan). Thin sections (80 nm thickness) were prepared using an ultramicrotome (Ultracut UCT, Leica Microsystems), collected on copper grids, stained with 2% uranyl acetate and lead citrate, and examined with a transmission electron microscope (JEM-1400Plus, JEOL Ltd., Japan).

## Results

### Growth rate and final cell length are enhanced in tetraploid Arabidopsis seedlings

**Fig 1:**
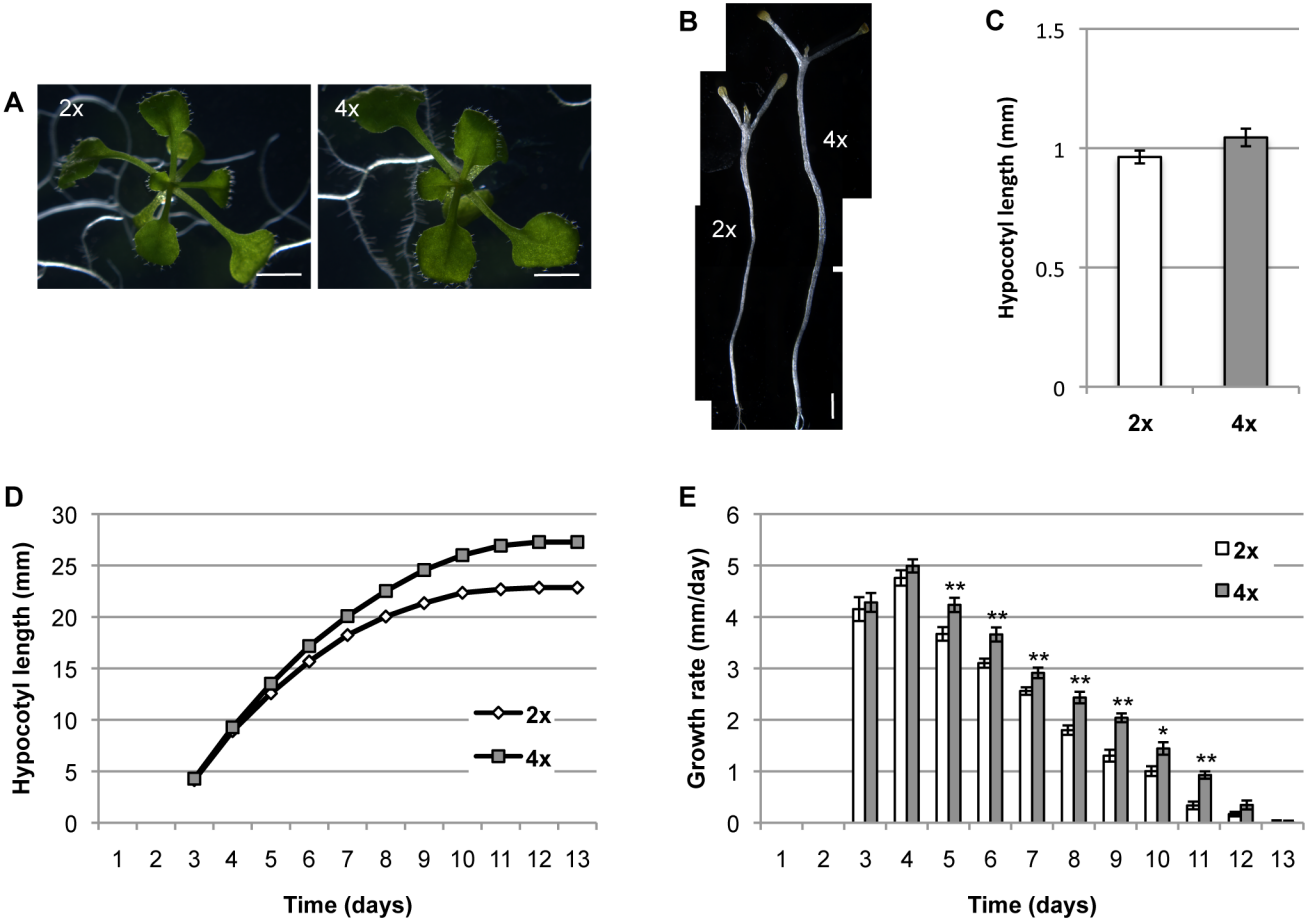
Growth of diploid and tetraploid hypocotyls. (A, B) Morphology of 13-day-old light- (A) and dark-grown (B) seedlings. Bars = 2 mm. (C) Hypocotyl lengths of 13-day-old light-grown seedlings. Error bars represent SE (n = 20). (D, E) Hypocotyl lengths (D) and growth rates (E) of dark-grown seedlings. Error bars represent SE (n = 15). Where absent, error bars are smaller than the symbols. Asterisks indicate a significant difference between the diploid and tetraploid plants (**p < 0.01, *p < 0.05, Student’s *t*-test). 2x, diploid plants; 4x, tetraploid plants.

To investigate the growth effects of increased ploidy in *A*. *thaliana*, we compared hypocotyl growth between diploid (wild-type) plants and tetraploid plants. In light-grown plants, no significant difference in overall morphological appearance was observed between diploid and tetraploid plants in either the hypocotyl or rosette leaves at various growth stages (Fig 1A and 1C), as reported previously [17]. By contrast, from day 5 onwards, hypocotyl length in dark-grown plants (Fig 1B and 1D) was significantly longer in tetraploid plants than in diploid plants. In addition, for days 5–11, the increment of hypocotyl length per day in dark-grown plants was higher in tetraploid than in diploid plants. This suggests that the promotion of hypocotyl elongation in tetraploid plants occurred as a result of a higher elongation rate in these plants compared to diploid plants (Fig 1E).

No significant differences in hypocotyl length were observed between diploid and tetraploid plants when other environmental conditions such as high temperature [18] and low R/FR ratio [19] were examined (S1 Fig).

**Fig 2:**
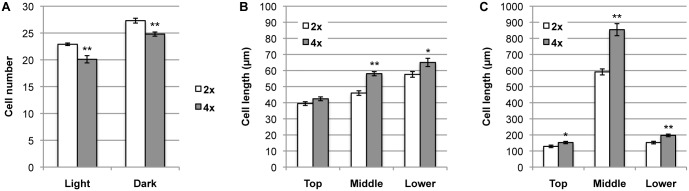
Cell numbers and lengths in diploid and tetraploid hypocotyls. (A) Total cell numbers for the whole hypocotyl of 5-day-old light- and dark-grown seedlings. Error bars represent SE (n = 10). (B, C) Cell lengths in top, middle, and lower hypocotyl regions of 5-day-old light- (B) and dark-grown (C) seedlings. Error bars represent SE (n = 30 cells from ten plants). Asterisks indicate a significant difference between the diploid and tetraploid plants (**p < 0.01, *p < 0.05, Student’s *t*-test). 2x, diploid plants; 4x, tetraploid plants.

To determine whether the promotion of hypocotyl elongation in tetraploid plants was due to an increase in cell number or to an increase in cell length, total cell numbers along the hypocotyl were counted, and average cell lengths were determined for the top, middle, and bottom hypocotyl regions. Total cell number in tetraploid plants was 90% of the cell number in diploids in both the light- and dark-grown plants (Fig 2A). Cells in tetraploid plants were significantly longer than in diploid cells, particularly in the central region (Fig 2B and 2C). The tetraploid:diploid cell-length ratio was higher in the dark-grown plants than in the light-grown plants for the whole hypocotyl region (Table 1). In light-grown plants, hypocotyl lengths did not differ significantly between diploid and tetraploid plants (Fig 1C). In these plants, the increased cell elongation in tetraploid plants was probably offset by the reduction in cell number. However, in dark-grown plants, cell elongation was predominant and overcame the diminution in cell number.

**Table 1:** Tetraploid:diploid hypocotyl cell-length ratios.

	Light	Dark
Top	1.08	1.18
Middle	1.26	1.44
Lower	1.13	1.29

The ratios of cell-lengths between tetraploid and diploid in the top, middle and lower regions of hypocotyls derived from 5-day-old light- and dark-grown seedlings were calculated using the data shown in Fig 2.

### Genome-wide identification of genes differentially expressed between tetraploid and diploid plants

**Fig 3:**
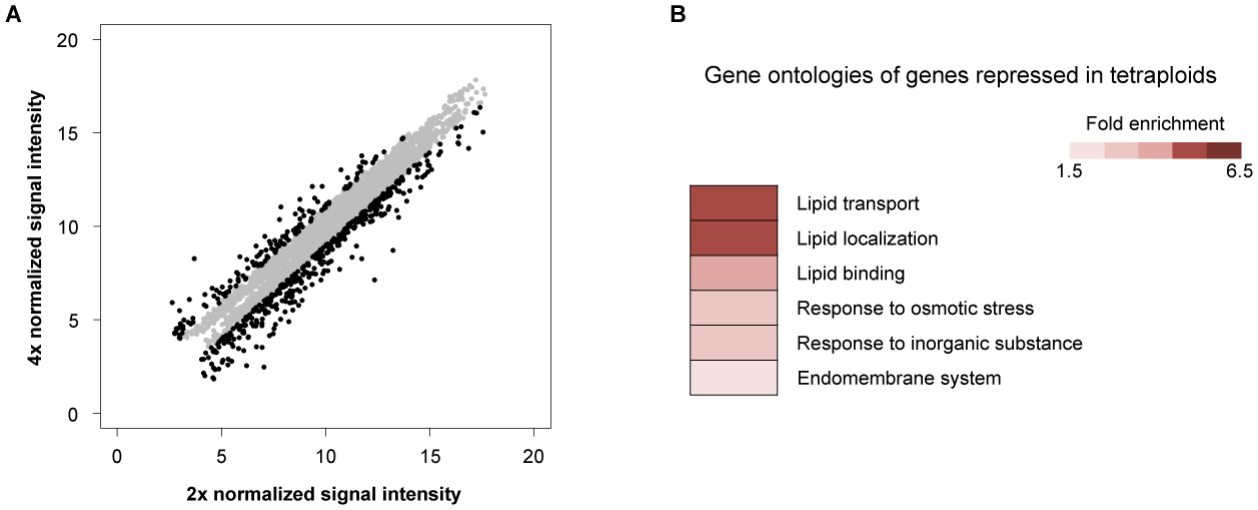
Global gene expression profiles in diploid and tetraploid dark-grown hypocotyls. (A) Scatter plot showing diploid and tetaraploid gene expression data in the apical growing region of 7-day-old dark-grown hypocotyls. Black dots represent genes whose expression levels differ by at least twofold between diploid and tetraploid. Gray dots represent genes below the twofold threshold. Genes with low signal-to-noise ratios were removed from analysis. (B) Significantly enriched Gene Ontology terms for genes whose expressions levels were at least twofold lower in tetraploid than in diploid plants. 2x, diploid plants; 4x, tetraploid plants.

Microarray transcriptome profiles of diploid and tetraploid plants were compared to identify ploidy-related genes involved in the determination of cell size. We noted that the cell elongation zone in the etiolated hypocotyl of Arabidopsis was restricted to the apical region (S2 Fig), as observed previously by Gendreau et al. [11]. Microarray analysis was therefore performed using total RNA obtained from the apical 4 mm region of 7-day-old etiolated hypocotyls. The analysis identified 416 genes that were differentially expressed between diploid and tetraploid plants using a significance threshold of FDR < 0.1 and a twofold differential cutoff (Fig 3A; S2 and S3 Tables). Approximately three-quarters (305) of the differentially expressed transcripts were more abundant in the tetraploid plants, and one quarter (111) was more abundant in diploid plants. Genes associated with three Gene Ontology (GO) terms (lipid transport, lipid localization and lipid binding) were enriched in the subset of genes repressed in tetraploid plants (Fig 3B). Five of these genes encode lipid transfer proteins (LTPs) (S4 Table), which are putatively implicated in cuticle formation [20]. Downregulated expression of these *LTP* genes in tetraploid plants was confirmed by real time RT-PCR analysis (S3 Fig). No enriched GO terms were found for genes upregulated in tetraploid plants, and no GO terms associated with plant hormones or cell wall metabolism were found in the upregulated or repressed gene sets in tetraploid plants.

### Differences in cuticular structure and function in tetraploid and diploid plants

**Fig 4:**
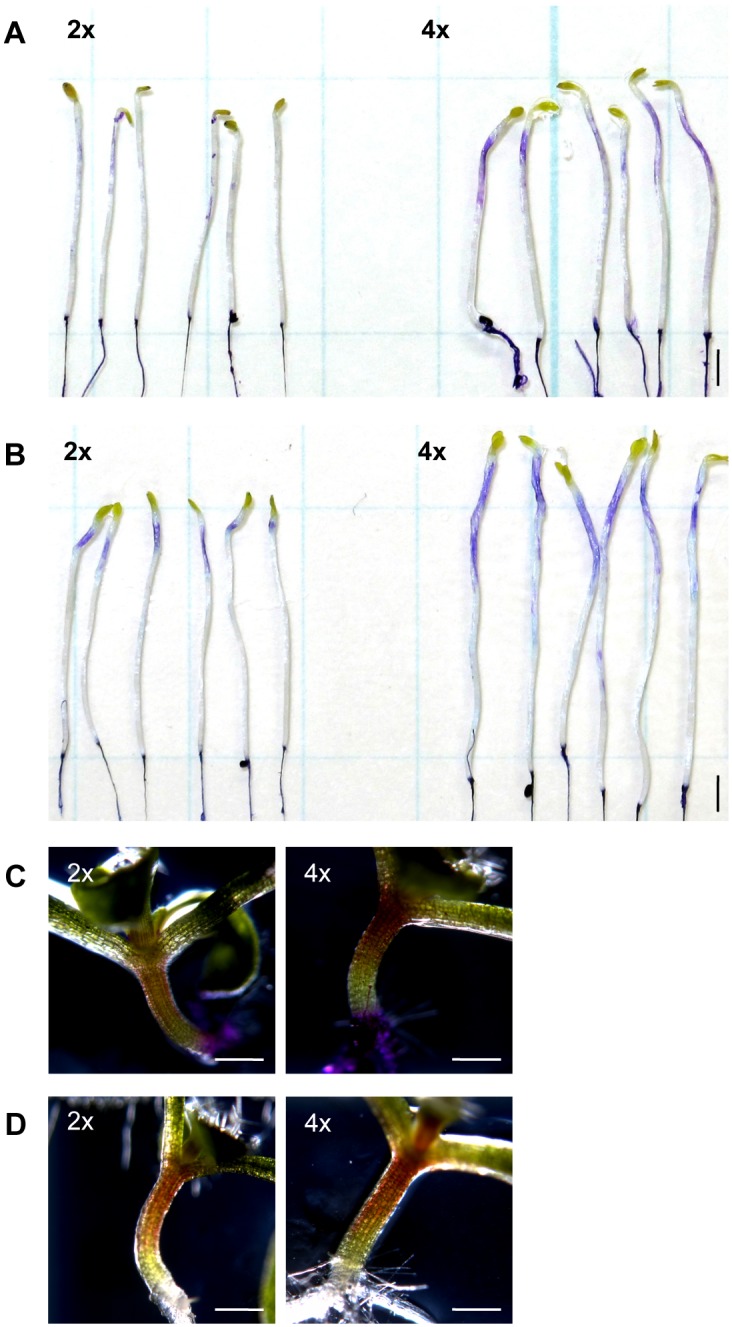
Cuticular barrier function in diploid and tetraploid hypocotyls as visualized by toluidine-blue staining. (A, B) 5-day-old (A) and 7-day-old (B) dark-grown seedlings stained with toluidine blue (TB). (C) 7-day-old light-grown seedlings stained with TB. (D) Unstained 7-day-old light-grown seedlings shown as control for Fig 4C. 2x, diploid plants; 4x, tetraploid plants. Bars = 2 mm (A, B), 500 μm (C, D).

The results of GO analysis implied that the structural and/or functional features of epidermal cuticle might be altered in tetraploid hypocotyls. Accordingly, we examined the chemical properties of the cuticle using toluidine-blue (TB). Penetrance of TB is indicative of defects in the barrier function of the cuticle against water and hydrophilic compounds [16]. Patterns of TB staining varied between diploid and tetraploid hypocotyls depending on the growth stage. In 5-day-old dark-grown seedlings, TB staining was not noticeable in the diploid hypocotyl but was apparent in the apical region of tetraploid hypocotyls (Fig 4A). In 7-day-old dark-grown seedlings, TB staining was restricted to the apical region in the diploid hypocotyls, whereas staining extended to subapical non-growing regions of the hypocotyl in tetraploid plants (Fig 4B). No TB staining was observed in light-grown hypocotyls, irrespective of ploidy level (Fig 4C and 4D). TB-stained regions on dark-grown hypocotyls largely correlated with the actively growing regions. It is possible that the altered cuticle barrier in dark-grown seedlings may reflect changes to structural features that are associated with an enhanced growth rate.

**Fig 5:**
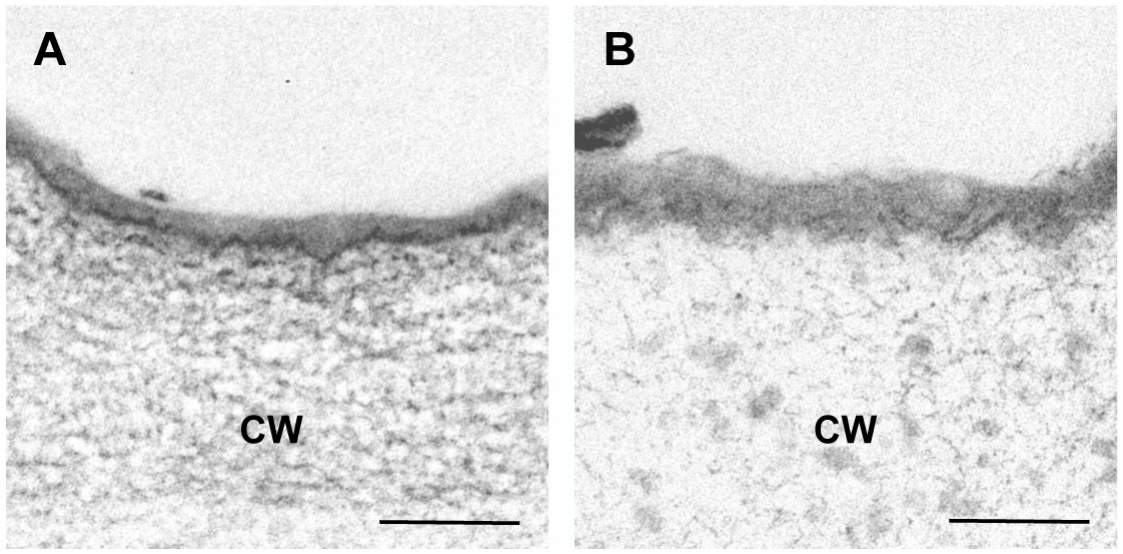
Cuticular structure in diploid and tetraploid dark-grown hypocotyls as assessed by transmission electron microscopy. Transmission electron micrographs of cuticle in epidermal cells derived from the apical 3 mm region of 7-day-old dark-grown hypocotyl of diploid (A) and tetraploid (B) seedlings. CW, cell wall; 2x, diploid plants; 4x, tetraploid plants. Bars = 100 nm.

Finally, using TEM, we examined the fine structure of the cuticle layer at the apical region of the hypocotyl in tetraploid and diploid seedlings. Osmium tetroxide staining revealed that the cuticular layer was more diffuse in tetraploid hypocotyls than in diploid hypocotyls, and was approximately 40% thicker in tetraploid than in diploid (Fig 5A and 5B). Similar alterations in the fine structure of the cuticle layer were observed in mutants defective in cuticle formation [21–23].

## Discussion

### Correlation between tetraploidy and cell size in Arabidopsis hypocotyl

The regulatory processes underlying cell-size determination in plants are still to be elucidated. Several previous studies noted a close correlation between cell size and ploidy level, particularly in epidermal cells of Arabidopsis rosette leaves and cotyledons [24, 25], but a causal link has not been firmly established, and this remains controversial [1, 12]. It is unclear whether a common mechanism for ploidy-dependent cell-size regulation would be shared between different plant species or between different tissue types.

In this study, we focused on a specific growth process: the development of Arabidopsis hypocotyls. Our results demonstrated that, compared to diploidy, tetraploidy enhanced both elongation rate and the final length of hypocotyl cells. We also noted that hypocotyl cell numbers were lower in tetraploid than in diploid seedlings. It should be noted that, although the promotive effect on cell length was more prominent in dark-grown seedlings, a small but significant length enhancement was observed even in light-grown seedlings.

The elevated elongation rate in the tetraploid hypocotyl became apparent as early as day 5 (Fig 1E). This demonstrated that the process underlying final cell size was active at the initiation of post-germination growth in the hypocotyl. This is supported by the observation that endoreduplication in Arabidopsis hypocotyls precedes the onset of rapid cell elongation growth in the root [26, 27].

Our data also showed that the minimal promotion of cell elongation in light-grown tetraploid Arabidopsis was offset by the reduction in cell number along the hypocotyl (Figs 1C and 2A; Table 1), and this masked the tetraploidy-induced growth promotion. Reduction of cell number in tetraploid Arabidopsis is a recognized phenomenon [28, 29] and is thought to occur as a result of slower DNA replication and cell division. Consequently, the enhancement of cell elongation by tetraploidy in Arabidopsis can only be observed under experimental conditions where the inhibitory effect of fewer cell divisions is overcome by the promotive effect on cell elongation.

Such phenomena have been attributed to compensation effect between cell number and cell size [30]. A recent genetic approach has elucidated that a certain type of compensation effect is induced when cell proliferation is impaired beyond a threshold level [31]. In the light of the concept of compensation effect, it makes sense that cell elongation is stimulated in the hypocotyls whose cell proliferation, as estimated by cell number, is reduced significantly in both the light- and dark-grown tetraploid hypocotyls (Fig 2). The more prominent stimulation of cell elongation in the dark-grown tetraploid hypocotyls, however, cannot be fully explained simply by the compensation effect, instead, might suggest the involvement of a process by which the cell-elongation can be promoted independently of reduction in cell-proliferation due to tetraploidy.

Previous research by Kurepa et al. [32] showed that a weak defect in the 26S proteasome led to an increase in the sizes of Arabidopsis organs, including the hypocotyl, as a result of increased cell expansion that compensated for a reduction in cell number. However, ploidy levels did not always increase by endoreduplication in all the cells of enlarged organs, and this indicated the involvement of factor(s) other than ploidy level in determining cell sizes.

### Roles of the cuticle in tetraploidy-dependent hypocotyl growth

The microarray data obtained in the present study are similar to those published previously [10, 17] and support the hypothesis that expression levels of the majority of individual genes remain mostly unaltered in high-ploidy plants. However, the present microarray analysis revealed that a small subset of genes was differentially expressed between tetraploid and diploid plants. A small number of the genes repressed in tetraploid plants compared to diploid seedlings were implicated in lipid-related functions as determined by GO analysis.

The cuticle, which covers the outer wall of the shoot epidermis in land plants, is a lipophilic layer composed of cutin and wax. Cutin is deposited on the outer surface of the polysaccharide-based cell wall layer, whereas wax is found within and on the surface of the cutin layer. Cutin is responsible for mechanical support and defense against pathogen attack [33], and the highly hydrophobic nature of the wax allows the cuticle to serve as a barrier against water loss. For cuticle deposition to occur, hydrophobic precursors of cutin and wax need to be transported from the sites of lipid synthesis in the plastid and endoplasmic reticulum and then progress through the hydrophilic cell wall to its outer surface. This biosynthetic flux is synchronized with the expansion of cell surface area during epidermal cell elongation to maintain a constant epicuticular wax load along the epidermis [34]. Some LTPs may be responsible for the transport of wax-polyester monomers across the cell wall, while ATP binding cassette (ABC) transporters from the ABCG subfamily are implicated in the transport across the plasma membrane [33].

LTPs are encoded by a large gene family that comprises at least 70 members in the Arabidopsis genome. Five members of this gene family, from the Type 1 and Type 5 LTP groups, have been implicated in cuticle formation [34]. LTP-GPI-ANCHORED 1 (LTPG1) and LTPG2, which are encoded by *Type 5 LTP* genes, can bind potential wax precursors and are required for the transport of wax to the cuticle [23, 35, 36]. LTPs are also involved in the defense response against pathogens. The *DIR1* gene encodes a Type 3 LTP. A loss of function mutant of *DIR1*, *dir1*, is defective in long-distance signaling in systemic resistance in Arabidopsis, suggesting that DIR1 interacts with lipid-derived signaling molecules and thereby mediates long-distance signaling [37].

In the present study, transcript levels of several genes encoding putative extracellular LTPs belonging to the Type 1, 4, and 5 LTP subfamilies were less abundant in the apical and subapical regions of dark-grown hypocotyls of tetraploid seedlings than in equivalent diploid tissues (S4 Table). As well as exhibiting enhanced cell elongation, the tetraploid hypocotyl exhibited a noticeable reduction in the barrier function of the epidermal cuticle compared to the diploid hypocotyl (Fig 4A and 4B). These results suggest that putative interaction pathways in Arabidopsis coordinate to control cell elongation and cuticle formation, and that these mechanisms interact to yield an elevated cell size in tetraploid compared to diploid hypocotyls.

Two possible models can explain the putative interaction pathways between cuticle formation and cell elongation. One model envisages the cuticle as a mechanical constraint that prevents or controls expansion growth of the whole hypocotyl. According to this model, a change in the mechanical properties of the epidermis would remove or reduce this growth constraint, resulting in the enhanced expansion growth in tetraploid hypocotyls. This model, however, is unlikely because mutants defective in cuticle formation do not always exhibit enhanced growth. The second model supposes that expansion growth of the hypocotyl stretches and damages the cuticle and, as a result, the cuticle needs to be continuously replenished by incorporation of new cuticular precursors into the cuticle layer. This model envisages that the compensation process is synchronized with expansion growth in the diploid hypocotyl but that growth rate exceeds the rate of replenishment in the tetraploid hypocotyl, resulting in the defective barrier function of the cuticle.

Very-long-chain fatty acids (VLCFAs), which are components of cuticular wax, are proposed to act as mediators or ligands that control gene transcription in bacteria, yeast, and mammals. In the Arabidopsis shoot apex, VLCFA synthesis in the epidermal tissue is thought to confine cytokinin synthesis to the vascular tissues and thereby restrict cell proliferation. This is supported by the observation that a reduction in VLCFA synthesis resulted in an increase in leaf size. These data suggested that shoot growth was controlled by interactions between the epidermis and the inner tissues of the plant body [38]. Any alteration or defect in cuticular organization at the epidermal surface of the tetraploid hypocotyl might affect the abundance or localization pattern of cuticular precursors, including VLCFAs, thereby removing growth constraints and enhancing cell expansion. The weakness of this model, however, is that it does not explain the mechanism by which growth promotion in tetraploids is initiated at the early stage of the hypocotyl growth. This question requires further investigation.

## Conclusions

The final hypocotyl length of *A*. *thaliana* (L.) Heynh. was longer in tetraploid than in diploid seedlings, particularly when plants were grown in the dark. The longer hypocotyl in tetraploid plants was a result of longer cell lengths in the tetraploid hypocotyl. To our knowledge, this is the first report to provide evidence that promotion of cell elongation is responsible for ploidy-dependent size determination in the Arabidopsis hypocotyl.

A group of genes implicated in the transport of cuticle precursors was significantly downregulated in the growing region of the tetraploid hypocotyl compared to the diploid hypocotyl. Furthermore, in these hypocotyl regions, cuticle permeability as estimated by TB staining and TEM imaging of cuticular structure differed between tetraploid and diploid seedlings.

Based on these findings, we propose that ploidy-dependent cell expansion in Arabidopsis hypocotyls is directly or indirectly related to cuticular function.

## Supporting Information

S1 Fig Hypocotyl lengths of diploid and tetraploid seedlings grown under a high temperature or low red/far red ratio conditions. Seedlings of diploid (2x) and tetraploid (4X) were grown for 13 days under white light (45 μmol m^-2^s^-1^) at 22°C (Control), under white light at 29°C (29°C) or under a low R/FR ratio (R/FR = 0.1) light conditions at 22°C (Low R/FR). Mean hypocotyl lengths with SE as vertical lines are shown (n = 20). There is no significant difference between the diploid and tetraploid plants as evaluated by Student’s *t*-test.
(TIF)

S2 Fig Growth in the elongation zone of diploid and tetraploid dark-grown hypocotyls. (A, B) Lengths in delimited regions of dark-grown hypocotyls of diploid (A) and tetraploid (B) seedlings. The upper 5-mm region of the hypocotyls, at ages of 3 to 10 days old, was divided into several ~1 mm subsegments by application of lanolin marks on the surface. After one day, the length between lanolin marks was measured. In both diploid and tetraploid, noticeable elongation was restricted to the upper ~1 mm throughout ages. (C) Growth rate on day (n) was defined as the increase in hypocotyl length from day (n-1) to day (n). Means with SE as vertical lines are shown (n = 10). Asterisks indicate significant difference between the diploid and tetraploid plants (**p < 0.01, *p < 0.05, Student’s *t*-test). 2x, diploid plants; 4x, tetraploid plants.
(TIF)

S3 Fig Expression levels of five *LTP* genes in tetraploid relative to diploid hypocotyls. Expression levels of five *LTP* genes (*At3g53980*, *At5g13900*, *At2g18370*, *At5g05960* and *At2g37870*) in the apical growing region of 7-day-old dark-grown hypocotyls were compared between diploid and tetraploid using quantitative real time RT-PCR using primers shown in S1 Table. Mean expression levels of four technical repeats in tetraploid plants, relative to diploid plants, are shown with SD. *α-tubulin* (*At1g04820*) expression level was used as a reference.
(TIF)

S1 Table Primer sets used for real time RT-PCR. (XLSX)

S2 Table Genes induced greater than twofold in tetraploid plants compared to diploid plants. Genes whose expression levels are twofold higher in the tetraploid plants compared to diploid plants are shown.
(XLSX)

S3 Table Genes repressed greater than twofold in tetraploid plants compared to diploid plants. Genes whose expression levels are twofold lower in the tetraploid plants compared to diploid plants are shown.
(XLSX)

S4 Table Repressed gene groups associated with lipid transport/localization/binding in tetraploid plants. (XLSX)
