# Supplementary figures and images for: Stimulation of Cell Elongation by Tetraploidy in Hypocotyls of Dark-Grown Arabidopsis Seedlings
Source: PLoS One. 2015 Aug 5;10(8):e0134547. doi: 10.1371/journal.pone.0134547 (PMC4526521; doi:10.1371/journal.pone.0134547)

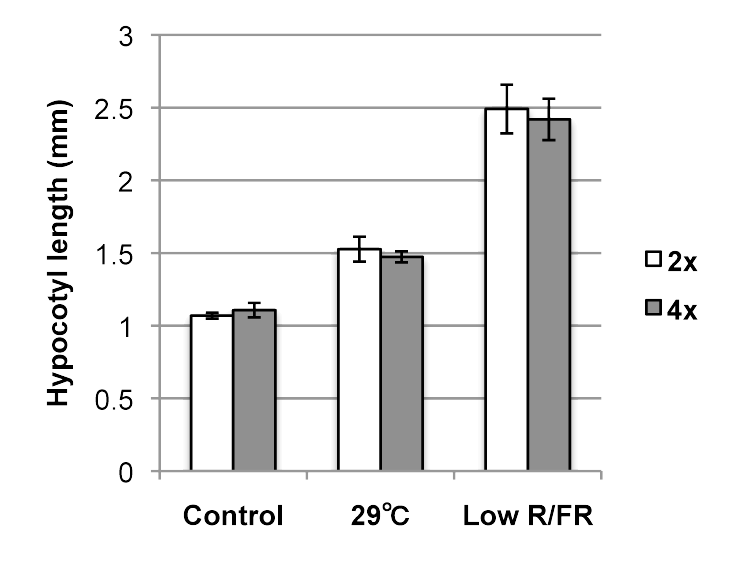

Supplement: S1 Fig — Seedlings of diploid (2x) and tetraploid (4X) were grown for 13 days under white light (45 μmol m-2s-1) at 22°C (Control), under white light at 29°C (29°C) or under a low R/FR ratio (R/FR = 0.1) light conditions at 22°C (Low R/FR). Mean hypocotyl lengths with SE as vertical lines are shown (n = 20). There is no significant difference between the diploid and tetraploid plants as evaluated by Student’s t-test. (TIF) [file pone.0134547.s001.tif]

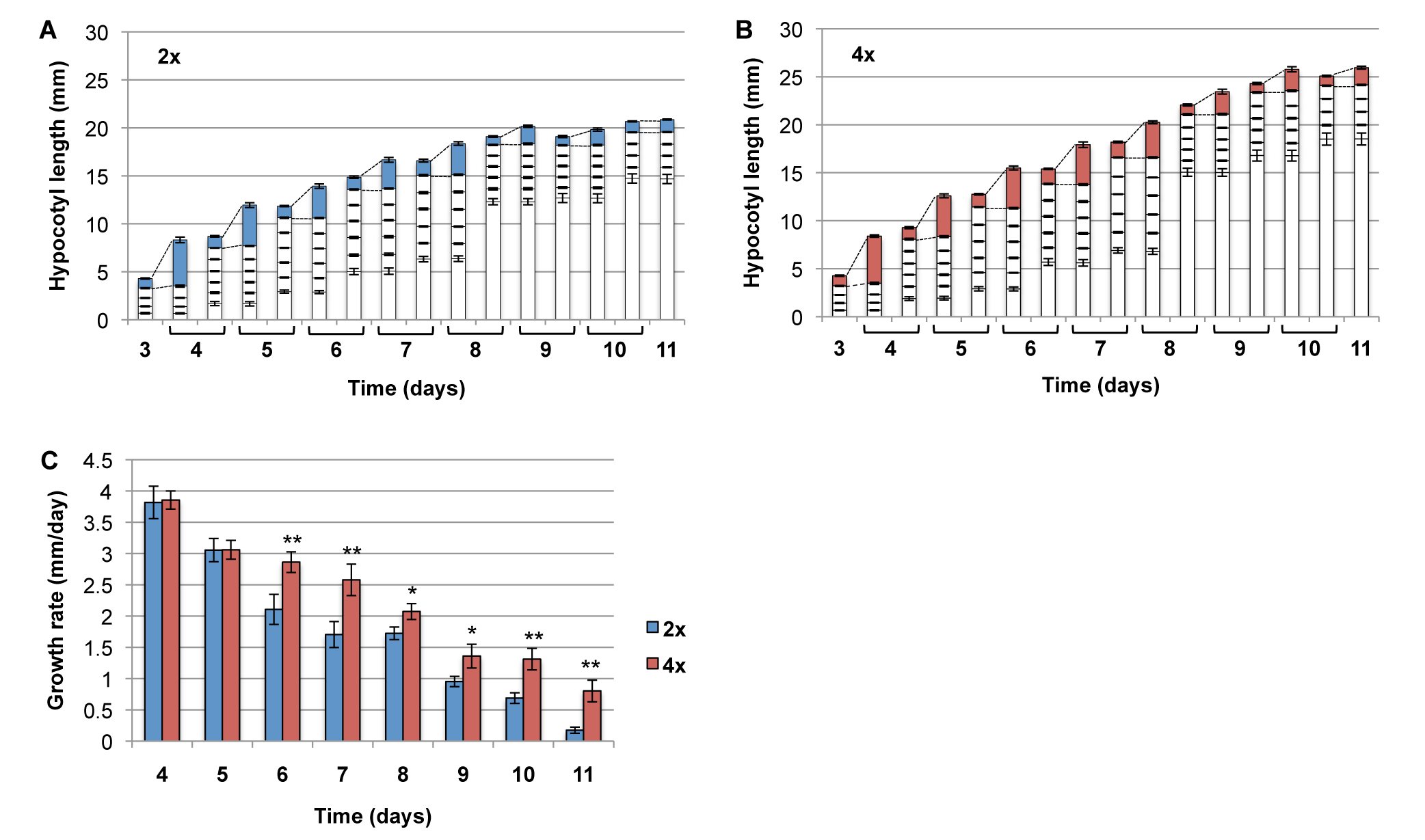

Supplement: S2 Fig — (A, B) Lengths in delimited regions of dark-grown hypocotyls of diploid (A) and tetraploid (B) seedlings. The upper 5-mm region of the hypocotyls, at ages of 3 to 10 days old, was divided into several ~1 mm subsegments by application of lanolin marks on the surface. After one day, the length between lanolin marks was measured. In both diploid and tetraploid, noticeable elongation was restricted to the upper ~1 mm throughout ages. (C) Growth rate on day (n) was defined as the increase in hypocotyl length from day (n-1) to day (n). Means with SE as vertical lines are shown (n = 10). Asterisks indicate significant difference between the diploid and tetraploid plants (**p < 0.01, *p < 0.05, Student’s t-test). 2x, diploid plants; 4x, tetraploid plants. (TIF) [file pone.0134547.s002.tif]

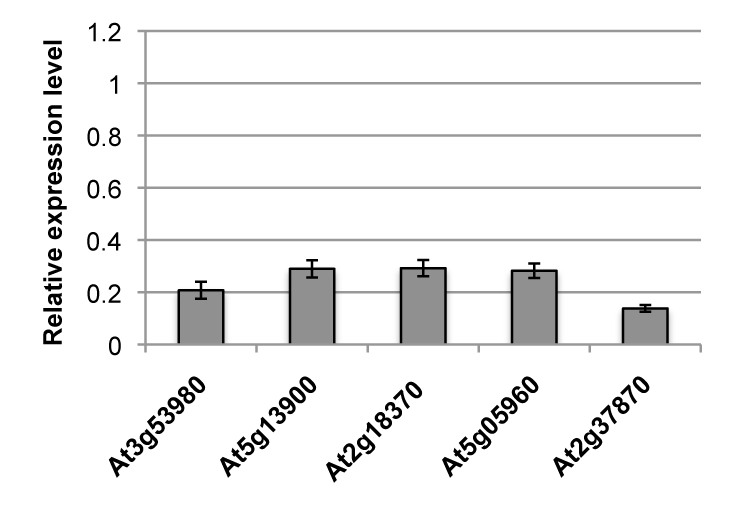

Supplement: S3 Fig — Expression levels of five LTP genes (At3g53980, At5g13900, At2g18370, At5g05960 and At2g37870) in the apical growing region of 7-day-old dark-grown hypocotyls were compared between diploid and tetraploid using quantitative real time RT-PCR using primers shown in S1 Table. Mean expression levels of four technical repeats in tetraploid plants, relative to diploid plants, are shown with SD. α-tubulin (At1g04820) expression level was used as a reference. (TIF) [file pone.0134547.s003.tif]
